# MicroRNAs in maxillofacial bone modeling and remodeling: implications for malocclusion development and orthodontic treatment

**DOI:** 10.3389/fcell.2024.1355312

**Published:** 2024-03-13

**Authors:** Baike Chen, Yuxin Zhang, Ousheng Liu

**Affiliations:** Hunan Key Laboratory of Oral Health Research & Hunan 3D Printing Engineering Research Center of Oral Care and Hunan Clinical Research Center of Oral Major Diseases and Oral Health and Xiangya Stomatological Hospital and Xiangya School of Stomatology, Central South University, Changsha, Hunan, China

**Keywords:** miRNA, orthodontic treatment, bone modeling, bone remodeling, maxillofacial bone, malocclusion

## Abstract

Modeling and remodeling are essential processes in the development and refinement of maxillofacial bones. Dysregulated bone modeling during the developmental stage may lead to maxillofacial bone malformations and malocclusion. Bone remodeling under mechanical loading serves as the biological basis for orthodontic treatment. Although previous reviews have indicated the significance of microRNAs (miRNAs) in bone metabolism, their roles in orchestrating maxillofacial bone modeling and remodeling remain unclear. This review aims to discuss the mechanisms by which miRNAs regulate the morphogenesis and development of maxillofacial bones, as well as their implications for maxillofacial malformations and malocclusion. Moreover, miRNAs participating in maxillofacial bone remodeling and their impacts on cell mechanosensing are also summarized. Given the intricate interplay of cells and signaling pathways, exosomal miRNAs emerge as the orchestrators of the modeling and remodeling processes. The diagnostic and therapeutic potentials of miRNAs are also highlighted in this review for future clinical applications.

## 1 Introduction

Maxillofacial bone malformation and malocclusion are associated with many congenital and postnatal malformations, including abnormalities in the size, shape, and alignment of maxillofacial bones and teeth (Dixon et al., 2011). The causes of such malformations and misaligned teeth (malocclusion) are complex. While some cases are associated with specific gene mutations (such as DiGeorge syndrome) (Cohen et al., 1999), most cases are non-syndromic and influenced by multiple factors (Dixon et al., 2011). The dysregulation of maxillofacial bone modeling and remodeling during different stages is considered to be the main cause of such malformations and malocclusions.

**FIGURE 1:**
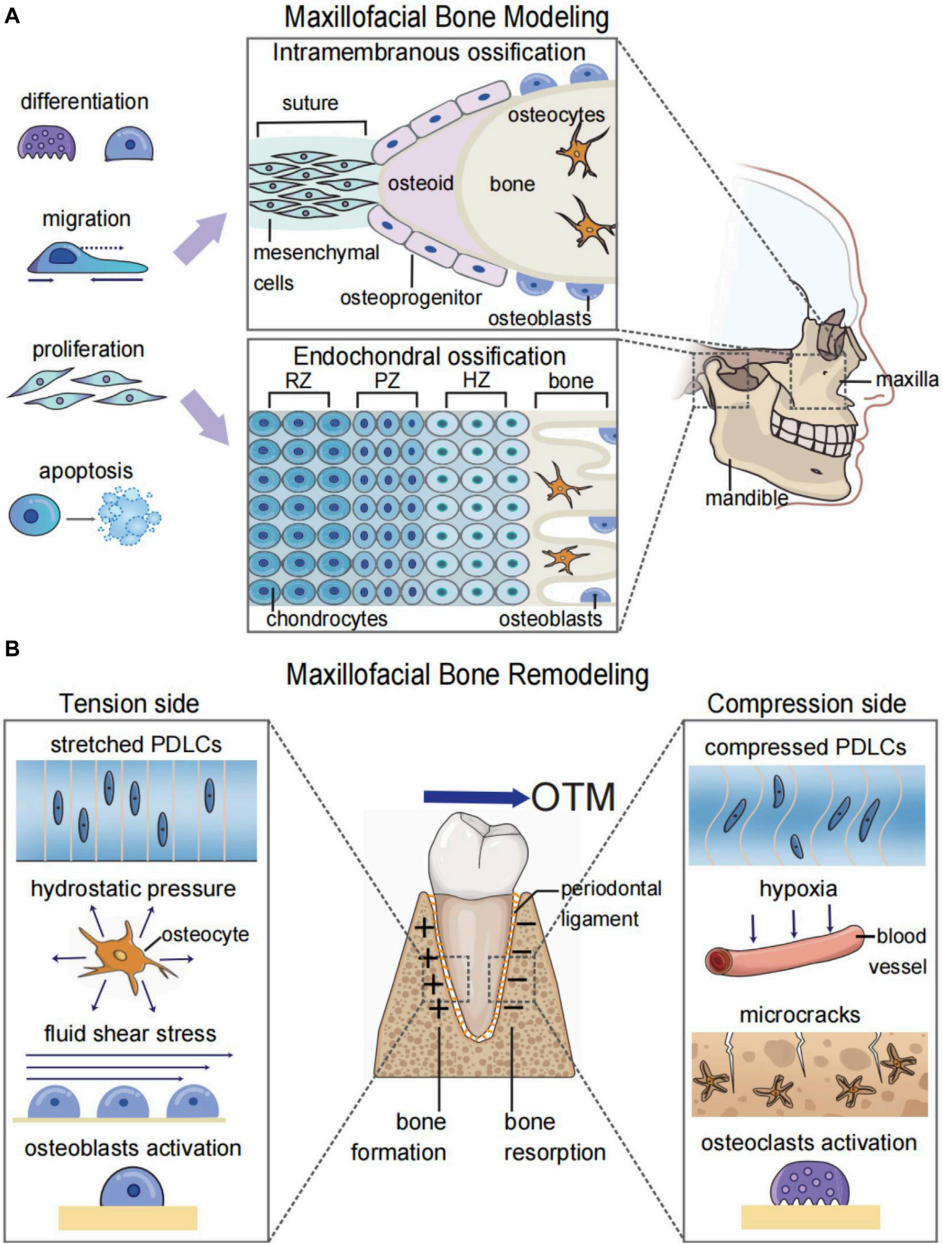
Graphic illustration of maxillofacial bone modeling and remodeling. **(A)** Maxillofacial bone modeling depends on the differentiation, migration, proliferation, and apoptosis of neural crest cells (NCCs) for the onset of ossification centers. Two types of ossification centers contribute to maxillofacial bone modeling: endochondral ossification for parts of the temporal bone and mandibular condyle and intramembranous ossification for the remaining maxillofacial bones. **(B)** Maxillofacial bone remodeling during OTM depends on the mechanosensitivity of both bone and soft tissue. The orthodontic force leads to the alteration of biomechanical microenvironments and the hypoxic area in the periodontal ligament and bone matrix. This results in osteoblastogenesis on the tension side and osteoclastogenesis on the compression side to realize the bodily movement of teeth. RZ, resting zone; PZ, proliferative zone; HZ, hypertrophic zone; OTM, orthodontic tooth movement; PDLCs, periodontal ligament cells.

Modeling and remodeling are different biological processes of bones (Seeman, 2009). Maxillofacial bone modeling, also known as maxillofacial bone development, refers to the changes in the size and shape of the maxillofacial bones. This process is predominant during body growth and is involved in the morphogenesis and increase of bone volume (Figure 1A) (Seeman, 2009). Maxillofacial bone remodeling is the process of renewing and refining the maxillofacial bones. Unlike bone modeling, which occurs during the developmental stage, bone remodeling is a lifelong process that involves the removal of old bone by osteoclasts and the formation of new bone by osteoblasts (Robling and Turner, 2009). Bone remodeling under mechanical forces is the biological foundation for orthodontic treatment (Li et al., 2021). Because of the mechanosensitivity of bones, orthodontists could apply forces to malpositioned teeth or bones for the treatment of malocclusion and maxillofacial bone malformation (Figure 1B).

In addition to the bone itself, soft tissues are another important participant during orthodontic tooth movement (OTM) and maxillofacial bone remodeling (Li et al., 2021), which are often overlooked when discussing systemic bone remodeling (Robling and Turner, 2009). OTM requires the responses of periodontal tissue toward mechanical stimulation. Without the periodontal ligament, ankylosed teeth or titanium implants cannot move under mechanical force (Humerfelt and Reitan, 1966; Li et al., 2021). Bone remodeling during the maxillary expansion process relies on structures such as the periosteal membrane and bone suture, which contain periosteal cells and fibroblasts (Murray and Cleall, 1971). Therefore, studying the synergistic effects between bone and soft tissue can improve our understanding of maxillofacial bone remodeling, thus enhancing the diagnosis and treatment of diseases.

Recent studies have indicated that microRNAs (miRNAs) are critical modulators in bone physiology (Wang et al., 2018; Brito et al., 2023). The dysregulation of miRNAs may affect maxillofacial bone modeling by regulating cell proliferation, migration, differentiation, and apoptosis (Suzuki et al., 2019b; Zhang W. et al., 2020b; Stüssel et al., 2021). During the remodeling process, miRNAs function as sensors of mechanical stimulation, regulators of cell behavior, and communicators between cells (Lv et al., 2020; Wu et al., 2020). Recent studies have also revealed the therapeutic potential of miRNAs. For example, miRNAs can be used as biomarkers for disease detection and to accelerate bone remodeling and tooth movement (Yu et al., 2017; Jiang et al., 2021). These findings enhance our understanding of the regulatory role of miRNAs in bone physiology and reveal prospects for their future clinical application.

## 2 The biogenesis and functions of miRNA

**FIGURE 2:**
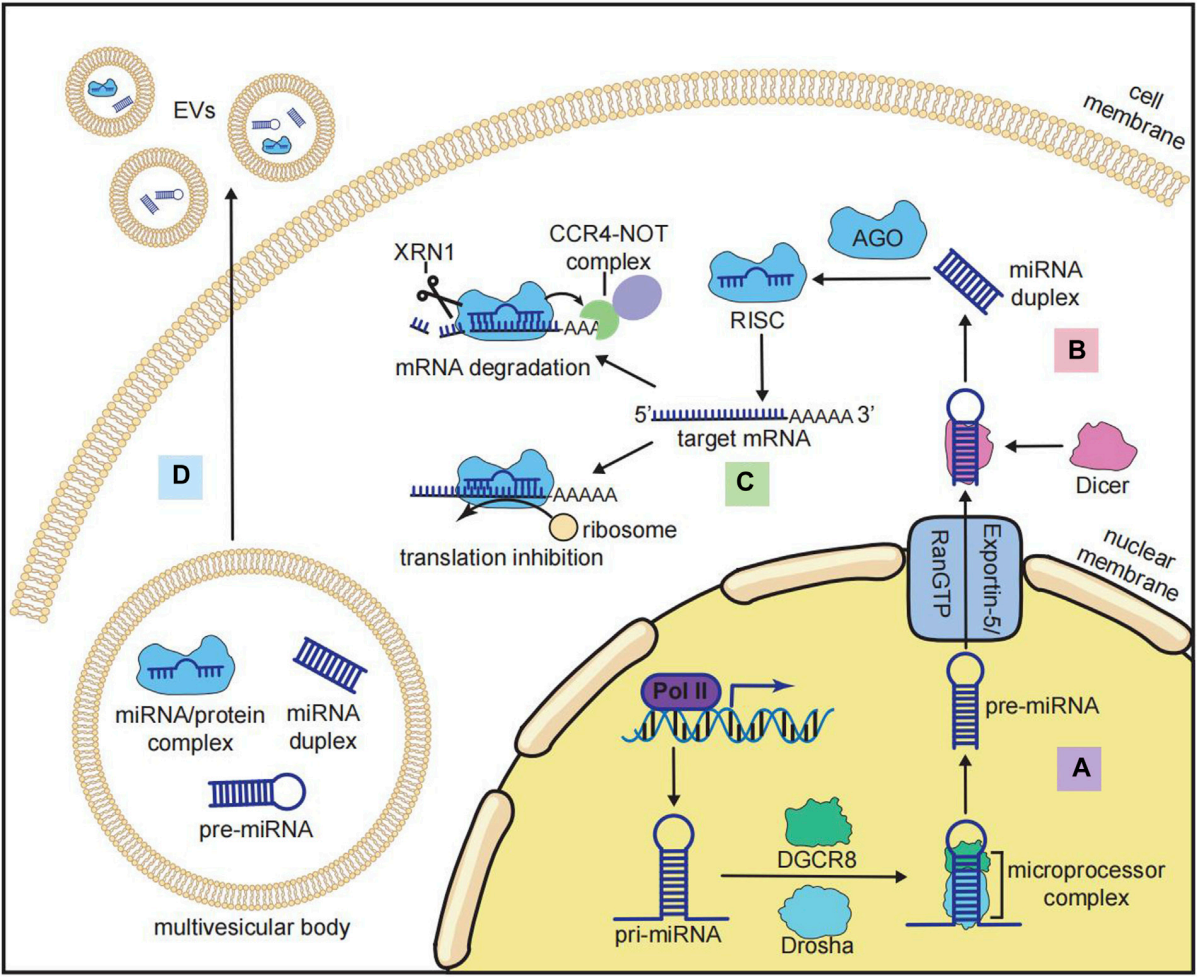
Biogenesis and functions of miRNAs. **(A)** Nuclear processing of miRNA: pri-miRNA is transcribed by RNA polymerase II (Pol II) and cropped by the microprocessor complex that consists of Drosha and DiGeorge syndrome critical region 8 (DGCR8) to become pre-miRNA. **(B)** Cytoplasmic processing of miRNA: after being transported from the nucleus to the cytoplasm by the exportin-5/RanGTP complex, pri-miRNA is cleaved by *Dicer* to become a mature miRNA duplex. **(C)** Inhibitory mechanisms of the RNA-induced silencing complex (RISC): mature miRNA is loaded by Argonaute (AGO) to form the RISC, and the RISC binds to the target mRNA at the 3′-untranslated region (3′-UTR) to recruit the CCR4-NOT complex and XRN1 for mRNA degradation or inhibiting the translation by ribosomes. **(D)** miRNA in extracellular vesicles (EVs): multiple forms of miRNA (pre-miRNA, mature miRNA, and miRNA/protein complex) could be incorporated into multivesicular bodies and secreted for intercellular communication.

miRNAs have a length of approximately 22 nucleotides that function as post-transcriptional modulators (Ha and Kim, 2014). miRNAs regulate gene expression by binding to their target mRNAs in a sequence-specific manner. The canonical biogenesis of miRNAs involves (i) transcription and generation of miRNA precursors in the nucleus (Figure 2A) and (ii) transportation and further processing of the precursors in the cytoplasm (Figure 2B) (Ha and Kim, 2014). miRNA genes are located in both introns and exonic regions of the genome. The loci of some miRNAs are spatially proximate to each other, such as the miR-17-92 cluster, which is generally co-transcribed and functions in a synergistic manner (Ries et al., 2017). The transcription of miRNAs is regulated by multiple transcription factors (TFs) and transcribed by RNA polymerase II into primary miRNAs (pri-miRNAs), which have a stem–loop structure and a length of over 1 kb (Ha and Kim, 2014). After transcription, long pri-miRNAs are further cropped into 60-nucleotide hairpin-shaped precursor miRNAs (pre-miRNAs). The cleavage of pri-miRNAs is mediated by a microprocessor complex consisting of Drosha (an RNase III-type endonuclease that crops dsRNAs) and DiGeorge syndrome critical region 8 (DGCR8) (Ha and Kim, 2014). pre-miRNAs are then transported from the nucleus into the cytoplasm by the exportin-5/RanGTP complex. pre-miRNAs in the cytoplasm are further cleaved by another RNase III-like endonuclease, Dicer, to become mature miRNA duplexes. These duplexes contain the miRNA-5p strands generated from the 5′ end and the miRNA-3p strands generated from the 3ʹ end of the pre-miRNAs (Ha and Kim, 2014).

The function of miRNAs is mediated by the miRNA-induced silencing complex (miRISC), in which the mature miRNA duplex is loaded with Argonaute (AGO) family proteins. The strand with lower 5′ stability is further selected and incorporated with AGO to form the miRISC (Treiber et al., 2019). The binding of miRISC to messenger RNAs (mRNAs) depends on the complementary sequences on the 3′UTR of mRNAs, which determine the target specificity of the miRISC (Treiber et al., 2019). The binding of mRNAs to miRISC results in both mRNA degradation (predominant mode) and translation inhibition (Figure 2C) (Eichhorn et al., 2014). miRNA-mediated mRNA degradation begins with the deadenylation of the poly(A) tail of mRNAs, and this process is mediated by the CCR4-NOT complex. The deadenylated mRNAs are further degraded by the 5′-to-3′ exonuclease XRN1 (Treiber et al., 2019). It should be noted that the binding of the miRISC and mRNAs does not require 100% complementarity of the sequences. One miRNA can bind to multiple mRNAs, and each mRNA can be targeted by many miRNAs (Treiber et al., 2019). In addition to mRNAs, other types of RNAs, such as lncRNAs, pseudogenes, and circRNAs, can also competitively bind to miRNAs and indirectly inhibit miRISC-mediated mRNA degradation (Zhang P. et al., 2019a). This interaction between different RNAs is known as the competing endogenous RNA (ceRNA) network, and it is crucial for investigating miRNA-mediated gene repression (Zhang P. et al., 2019a).

Furthermore, miRNAs can function in intercellular communication on a larger scale. Numerous miRNAs can be encapsulated by extracellular vesicles (EVs), such as exosomes, and transported through cells, tissues, and organs (Figure 2D) (Groot and Lee, 2020). The sorting and enrichment of miRNAs into exosomes is an active process mediated by miRNA-binding proteins like AGO, nSMase2, or the intrinsic characteristics of miRNAs (Groot and Lee, 2020). As a result, exosomal miRNAs are now considered important regulators of bone metabolism.

## 3 miRNAs in maxillofacial bone modeling and malocclusion development

During the embryonic stage, cells originating from the neural crest (neural crest cells, NCCs) combine with undifferentiated epithelial cells to form five facial processes that are the prototypes of the facial system (Dixon et al., 2011). The fusion of these processes determines facial formation and is ensured by appropriate miRNA levels in a temporal and spatial manner (Seelan et al., 2022). The dysregulation of miRNAs has been linked to congenital orofacial defects, such as cleft lip/palate. These defects can further lead to maxillofacial bone malformation and malocclusion (Dixon et al., 2011). Iwaya et al. summarized how miRNAs participate in the development of cleft lip/palate (Iwaya et al., 2023). In summary, miRNAs participate in the fusion of facial processes by regulating the migration (Stussel et al., 2022), proliferation (Suzuki et al., 2019b), and apoptosis (Zhang W. et al., 2020b) of NCCs and epithelial cells (shown in Table 1). miRNAs can also be used as biomarkers for cleft lip/palate. The salivary or plasma levels of some miRNAs (such as miR-141, miR-223, and miR-324-3p) are different between non-syndromic orofacial cleft patients and normal individuals, making them useful for clinical diagnosis (Li J. et al., 2016b; Grassia et al., 2018).

**TABLE 1:** miRNAs in maxillofacial bone development and malformations.

microRNAs	Species/cell types	Targets	Biological effects	Reference
miR-374a, miR-4680, and miR-133b	Human palatal mesenchymal cells	*FGFR1, FGFR2, WNT5A, FGF9, TP63,* and *TBX1*	Inhibits human embryonic palatal cell proliferation	Suzuki et al. (2019a)
miR-200b	Mouse embryos	*Smad2* and *Snail*	Inhibits EMT and palatal fusion	Shin et al. (2011)
miR-124-3p	mouse embryonic lip mesenchymal cells (mELM cells)	*Bmpr1a, Cdc42, Pbx3,* and *Tgfbr1*	Inhibits cell proliferation in mELM cells	Suzuki et al. (2019b)
miR-655-3p and miR-497-5p	Human lip fibroblasts	Multiple CL/P genes	Inhibits cell proliferation in human lip fibroblasts	Gajera et al. (2019)
miR-149	Human neural crest cells (hNCCs)	-	Promotes hNCC migration	Stussel et al. (2022)
miR-16-2-3p	Mouse palatal mesenchymal cells (MPMCs)	*PDPK1*	Increases cleft lip tissues, inhibits MPMC proliferation and migration and induces apoptosis	Han et al. (2019)
miR-106a-5p	Mouse palatal mesenchymal cells	*Tgfbr2*	Increases cleft palatal tissues and induces apoptosis in mouse palatal mesenchymal cells	Zhang et al. (2020b)
miR-21	miR-21	-	Reduces mandibular molar and alveolar bone size	Schwarze et al. (2021)
	Knockout (KO) mice			
miR-26b	miR-26b OE mice	*Lef-1*	Inhibits tooth development with maxillofacial defects	Eliason et al. (2020)
miR-17-92 cluster	miR-17-92 conditional knockout (CKO) mice	-	Reduces alveolar bone size and tissue volume	Ibrahim et al. (2016)
miR-17-92 cluster	Plasmid-based miRNA inhibition system (PMIS)–miR-17-92 mice	*Tgfbr2*	Inhibits palatogenesis with maxillofacial defects	Ries et al. (2017)
miR-200c	PMIS–miR-200c mice	*Sox2* and *Klf4*	Inhibits ossification of maxillofacial bones	Akkouch et al. (2019a)
miR-30d-5p	Mandibular prognathism (MP) patients	-	Is increased in mandibular bone tissues of MP patients	Tian et al. (2015)
let-7i-3p and miR-595	MP patients	-	Increased let-7i-3p levels and decreased miR-595 levels in the serum sample of MP patients	Gu et al. (2014)

**FIGURE 3:**
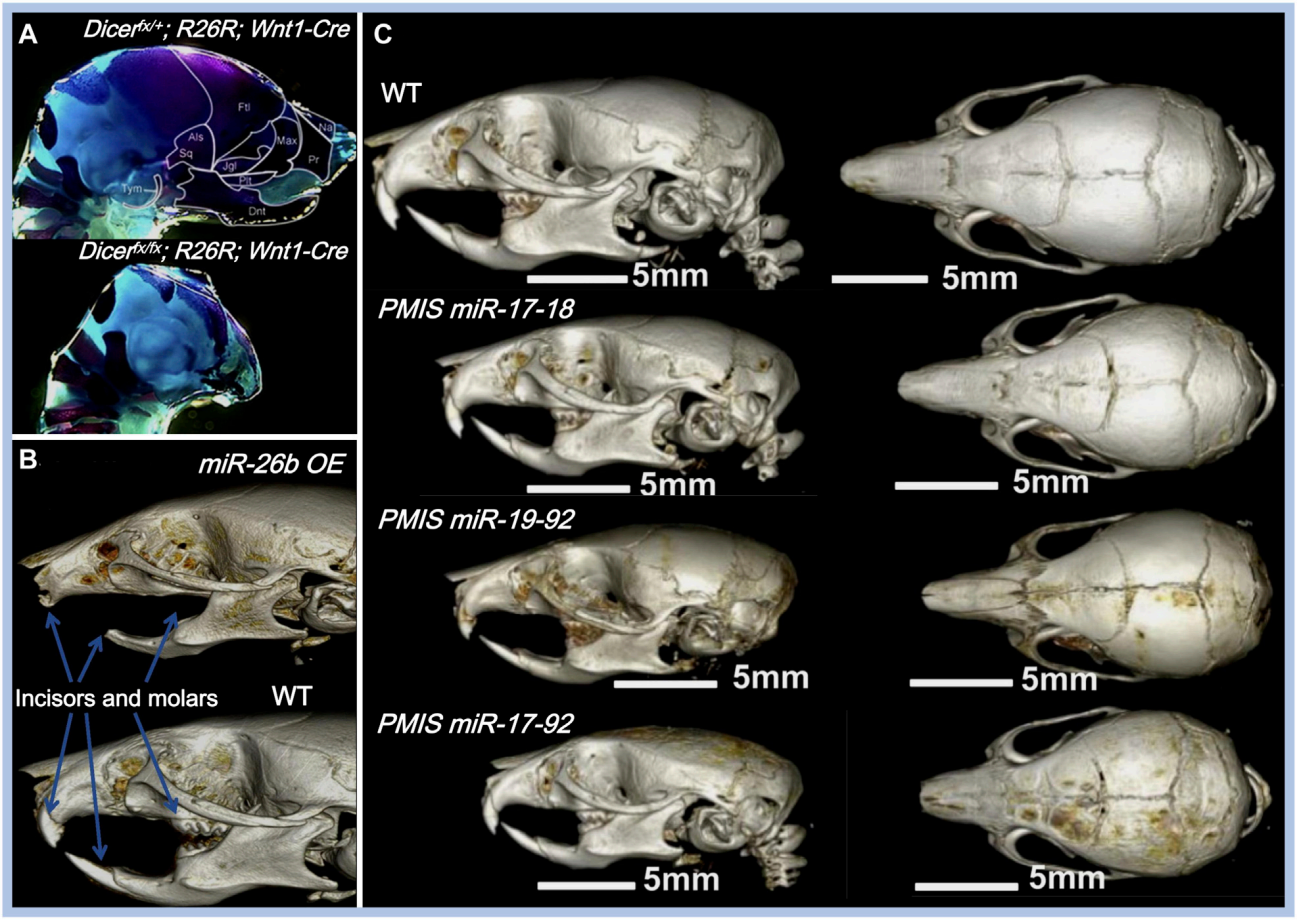
Effects of miRNAs on maxillofacial bone malformation. **(A)** Conditional knockout of *Dicer* in *Wnt1-*expressing NCCs abolished the development of NC-derived maxillofacial bones during the embryonic stage. Copyright 2010, Elsevier. **(B)** Effects of inhibiting *miR-17-18, miR-19-92,* and *miR-17-92* with the plasmid-based miRNA inhibition system (PMIS) on maxillofacial bones. Abnormal sizes of maxillofacial bones are distinct in all PMIS groups. Copyright 2021, Elsevier. **(C)** Mice overexpressing *miR-26b* (*miR-26b OE*) displayed anodontia (both molars and incisors) and shorter mandibular length. Copyright 2020, Frontiers.

Apart from their roles in the fusion of facial processes, miRNAs also regulate the growth pattern, size, and shape of the maxillofacial bones (shown in Table 1). The conditional knockout of *Dicer*, which is involved in the cleavage of pre-miRNAs, results in overall miRNA dysregulation and severe maxillofacial malformations, including maxillofacial hypoplasia, microstomia, an underdeveloped palate, and a short nose (Figure 3A) (Huang et al., 2010; Zehir et al., 2010; Nie et al., 2011). Neural crest-derived structures are mostly affected in *Dicer* knockout mice. Bones derived from NCCs such as nasal, palatine, maxillary, and mandibular bones remain undeveloped, while mesoderm-derived bones such as the parietal bones are not affected (Huang et al., 2010). Moreover, tooth enamel derived from dental epithelial cells is also absent in *Dicer* knockout mice (Huang et al., 2010). *Dicer* is essential for the survival of the differentiating NCCs but not for the migration of NCCs. The lack of *Dicer* leads to massive apoptosis of the differentiating NCCs and inhibits bone formation (Zehir et al., 2010; Nie et al., 2011). NCC-specific disruption of *Dicer* inhibits cartilaginous tissue formation in the first pharyngeal arch (PA1)-derived structures, such as maxillary and mandibular bones (Sheehy et al., 2010). The disruption of *Dicer* reduces *Dlx2* expression in PA1-derived structures, which regulates NCC patterning during PA1 development (Sheehy et al., 2010). *Dicer* knockout inhibits *Dlx2* expression, possibly through miR-452 and downstream *Wnt5a* levels. Targeting miR-452 abolishes the development of PA1-derived mandibular components with increased *Wnt5a* levels, thereby inhibiting the EMT of NCC-derived mesenchyme in PA1 (Sheehy et al., 2010).

*Dgcr8*, located on human chromosome 22q11, is another gene involved in miRNA biogenesis. *LgDel* mice with *Dgcr8* deletion show congenital maxillofacial anomalies, which are similar to those observed in humans with DiGeorge syndrome, a disease with congenital maxillofacial and cardiovascular defects (Cohen et al., 1999; Karpinski et al., 2013). The elevation of the palatal shelves in *LgDel* mice fails to occur during fetal development. Furthermore, the mandible size of postnatal *LgDel* mice is smaller than that of wild-type (WT) mice, resembling the mandibular anomalies observed in DiGeorge syndrome patients (Karpinski et al., 2013). It is reported that approximately 40% of DiGeorge syndrome patients have a cleft palate and other facial anomalies associated with overall miRNA dysregulation (Cai et al., 2014), indicating that miRNA biogenesis is closely associated with maxillofacial bone malformation.

Some miRNAs affect maxillofacial bone malformation and malocclusion in mice. The *miR-21* knockout mice have smaller mandibular molars and alveolar bone size (Schwarze et al., 2021), suggesting that miR-21 can affect tooth morphogenesis. *miR-26b* overexpression is associated with anodontia (both molars and incisors) and shorter mandible, nasal bone, parietal bone, frontal bone, and cranial base lengths in mice (Figure 3B) (Eliason et al., 2020). Compared with WT mice, the cranial base angle and ramus height of *miR-26b* overexpressing mice are decreased (Eliason et al., 2020). *Lef-1*, the marker of early tooth and maxillofacial development, is inhibited by miR-26b. The upregulation of miR-26b coincides with the decrease in *Lef-1* levels in the early stages of dental epithelial development *in* *vivo*, which is important for incisor growth (Eliason et al., 2020).

The miR-17-92 cluster (miR-17, miR-18a, miR-19a, miR-19b-1, miR-20a, and miR-92a) is a highly conserved miRNA cluster and key regulator during palatogenesis (Ries et al., 2017). The conditional knockout of the *miR-17-92* cluster in type-I collagen-expressing cells reduces alveolar bone size and increases periodontal ligament space in mice, making them more susceptible to orthodontic forces (Ibrahim et al., 2016). In another study, Ries et al. compared three different inhibitory complexes of *miR-17-92* (inhibition of *miR-17-18, miR-19-92,* and *miR-17-92*) using the plasmid-based miRNA inhibition system (PMIS) in mice. Alterations in maxillofacial bone size and arrested palatogenesis occurred in all groups (Figure 3C), which might be associated with elevated *Tgfbr2* levels and the Tgfβ signaling pathway (Ries et al., 2017). Similarly, the absence of miR-200c reduces the ossification of the maxilla and mandible in mice and inhibits the osteogenic differentiation of human bone marrow stem cells (hBMSCs) (Akkouch et al., 2019a). BMP and Wnt signaling were activated by miR-200c to promote the differentiation of ameloblasts and osteoblasts (Cao et al., 2013; Akkouch et al., 2019a).

Although animal development models have shown that certain miRNAs have regulatory effects, confirming the role of these miRNAs in human maxillofacial malformations and malocclusion is challenging. Using microarray analysis, Tian et al. identified 7 upregulated miRNAs and 11 downregulated miRNAs in the bone samples of skeletal class III malocclusion patients who underwent genioplasty (Tian et al., 2015). miR-30d-5p might have a potential regulatory function in the development of the mandible by inhibiting the expression of *Runx-2*, the transcription factor of osteoblasts (Tian et al., 2015). miR-30d-5p can also inhibit osteogenic differentiation *in vitro* (Wu Z. H. et al., 2018b). To further understand the correlation between miRNAs and mandibular prognathism (MP), another study compared the serum miRNA levels between MP patients and controls in different maxillofacial developmental stages (Gu et al., 2014). The upregulation of let-7i-3p and downregulation of miR-595 occur in MP patients at both the mixed dentition stage (aged 8–10 years) and the early permanent dentition stage (aged 10–14 years). Considering the importance of early treatment of MP before its growth peak, serum let-7i-3p and miR-595 can be utilized as potential non-invasive biomarkers for MP detection (Gu et al., 2014).

## 4 miRNAs in maxillofacial bone remodeling and orthodontic tooth movement

Maxillofacial bones are highly dynamic and undergo continuous remodeling throughout life. This process is achieved through the differentiation of osteoblasts and osteoclasts at different sites with the participation of the vascular, immune, and nervous systems (Li et al., 2021). miRNAs that function as the mechanosensors and communicators during these biological processes are summarized in this section.

### 4.1 Mechanosensitive miRNAs in bone remodeling

Mechanical sensing is the initial segment of the remodeling repertoire. Although many cell types can respond to mechanical stimulation, osteocytes in the bone matrix and fibroblasts in soft tissues (periodontal ligament, gingiva, and bone suture) are the primary mechanosensors (Li et al., 2021). The orthodontic force leads to the deformation of the extracellular matrix in both bone and soft tissue, resulting in altered fluid flow in the lacunar–canalicular system and increased hydrostatic pressure or fluid shear stress (FSS) of the interstitial fluid (Mak et al., 1997). Mechanical loading also leads to microcracks and hypoxia on the compression side of the alveolar bone, resulting in osteocyte apoptosis and bone resorption (Verna et al., 2004; Song et al., 2020). miRNAs that respond to this mechanical stimulation play key roles in regulating OTM and maxillofacial bone remodeling (shown in Table 2). They are involved in cell proliferation, differentiation, and migration by targeting multiple signaling pathways.

**TABLE 2:** Mechanosensitive miRNAs in cell mechanosensing, proliferation, and differentiation.

microRNAs	Mechanical loading	Cell type	Targets	Biological effects of miRNAs	Reference
miR-29b-3p↓	Strain	Osteocytes	*Igf-1*	Inhibits secretions of IGF-1 and osteoblast differentiation	Zeng et al. (2019)
miR-181b-5p↑	Strain	Osteocytes	*PTEN*	Promotes human periodontal ligament stem cell (hPDLSC) proliferation and osteogenic differentiation	Lv et al. (2020)
miR-21↑	Strain	hPDLSCs	*PDCD4*	Promotes hPDLSC osteogenic differentiation	Chen et al. (2016a)
miR-34a and miR-146a↓	Strain	hPDLSCs	*CELF3*	Inhibits hPDLSC osteogenic differentiation	Meng et al. (2021)
miR-195-5p↓	Strain	hPDLCs	*WNT3A*, *FGF2,* and *BMPR1A*	Inhibits hPDLC osteogenic differentiation	Chang et al. (2017)
miR-29↓	Strain	hPDLCs	*COL1A1, COL3A1,* and *COL5A1*	Regulates extracellular matrix (ECM) homeostasis	Chen et al. (2015)
miR-503-5p↓	Strain	rBMSCs	*Runx2* and *Alp*	Inhibits bone marrow stem cell (BMSC) osteogenic differentiation	Liu et al. (2017)
miR-103a↓	Strain	hFOB1.19	*RUNX2*	Inhibits osteogenic differentiation	Zuo et al. (2015)
miR-146b-5p↑	Strain	OCCM-30	*SMAD4*	Promotes cementoblastogenesis	Wang et al. (2016b)
miR-138↓	Strain	hBMSCs	*PTK2*	Inhibits BMSC osteogenic differentiation	Wu et al. (2018a)
miR-466d-5p and miR-466f-3p↑	Strain	rAECs	-	Regulates epithelial permeability	Yehya et al. (2012)
miR-191, -3070a↑ and miR-33 and -218↓	Strain	MC3T3-E1	-	Regulates osteogenic differentiation	Guo et al. (2015)
miR-218-5p, -138-5p, -221-3p, -132-3p↑ and miR-133a-3p, -145-3p, -143-5p, -486-3p, and -210-3p↓	Strain	hPDLCs	Hippo signaling pathway	Regulates osteogenic/cementogenic differentiation	Wu et al. (2019)
miR-494-3p↑	Compression	MC3T3-E1	*Fgfr2* and *Rock1*	Inhibits cell proliferation	Iwawaki et al. (2015)
miR-3198↑	Compression	hPDLCs	*OPG*	Promotes osteoclastogenesis	Kanzaki et al. (2019)
miR-26a↓	Compression	hPDLCs	*JAGGED1*	Inhibits osteoclastogenesis	Zhang et al. (2021)
miR-33-5p↑	Fluid shear stress (FSS)	MC3T3-E1	*Hmga2*	Promotes osteogenic differentiation	Wang et al. (2016a)
miR-132↑	FSS	hPDLCs	*mTOR*	Promotes hPDLC proliferation and osteogenic differentiation	Qi and Zhang (2014)
miR-140-5p↓	FSS	MC3T3-E1	*Vegfa* and *Erk5*	Inhibits osteoblast proliferation	Wang et al. (2021b)
miR-20a, -21, -19b, -34a, -34c, -140, and -200b↓	FSS	MC3T3-E1	-	Regulates osteogenic differentiation	Mai et al. (2013)
miR-125a-5p↑	Orthodontic force	hPDLCs	*ETV6*	Promotes M2 polarization	He et al. (2021b)
miR-21-5p↑	Unilateral anterior crossbite (UAC) model	Condylar chondrocytes	*Spry1*	Promotes angiogenesis and ECM degradation	Ma et al. (2020)

#### 4.1.1 Osteocytes

As the most abundant cell type in bones, osteocytes are considered the most important mechanosensors in bone remodeling (Matsumoto et al., 2013). Using a biaxial random positioning machine (RPM) to mimic microgravity, Chen et al. demonstrated that miR-15a, miR-221, and miR-29a are downregulated in MLO-Y4 osteocytes under the mechanical unloading conditions (Chen Z. et al., 2016b). Similarly, tensile strain downregulates miR-29b-3p in osteocytes, thereby increasing the secretion of IGF-1 and promoting osteoblast differentiation in a dose-dependent manner (Zeng et al., 2019). After being exposed to cyclic stretch, 121 miRNAs are upregulated, including miR-181b-5p, and 85 miRNAs are downregulated in osteocyte-derived exosomes compared to the unstimulated group. Stimulated exosomes exhibit the enhanced potential to promote the proliferation and osteogenic differentiation of human periodontal ligament stem cells (hPDLSCs) (Lv et al., 2020). The miR-181b-5p/PTEN/AKT signaling pathway, along with increased *BMP2/RUNX2* expression, might contribute to the enhanced osteogenic ability of stimulated osteocyte-derived exosomes (Lv et al., 2020). Although osteocytes are the housekeepers and major mechanosensors in bones, the study of mechanosensitive miRNAs in osteocytes is still lacking. Therefore, further studies should assess the role of osteocyte-derived miRNAs in OTM and bone remodeling.

#### 4.1.2 Fibroblasts

Fibroblasts, as the most abundant cell type in the periodontal ligament, periosteal membrane, and bone sutures, are considered to be the special mechanosensors in orthodontic treatment (Hou et al., 2007; Li et al., 2021). There is also a small population of fibroblast-like cells expressing CD146, CD73, CD90, and CD105 with multiple differentiation potentials, known as mesenchymal stem cells (MSCs), such as periodontal ligament stem cells (PDLSCs) and bone marrow stem cells (BMSCs), which are also considered to be mechanosensitive (Meng et al., 2021).

miR-21 is an important regulator during OTM. In *miR-21*-deficient (*miR-21−/−*) mice, the lack of *miR-21* significantly inhibits OTM and the differentiation of osteoblasts on the tension side compared to WT mice. This effect is likely due to the inhibition of programmed cell death 4 (*Pdcd4*) and elevated downstream *C-fos* levels (Chen N. et al., 2016a). During the expansion of the palatal sutures, *miR-21−/−* mice exhibit a slower migration rate and fewer periosteal cells in the expanding area. *miR-21* deficiency also inhibits the proliferation and migration rate of BMSCs *in vitro* (Li M. et al., 2020a). Several other miRNAs are also involved in the mechanically induced osteogenic differentiation of PDLCs. After FSS stimulation, the level of miR-132 is upregulated in hPDLCs, with increased proliferation and osteogenic differentiation of hPDLCs, while the knockdown of miR-132 leads to opposite outcomes (Qi and Zhang, 2014). miR-132 plays its regulatory role probably via the phosphorylation of P13K, AKT, and p70S6K proteins and the activation of the mTOR pathway (Qi and Zhang, 2014). Another study showed that the upregulation of miR-132 under microgravity inhibits osteoblast differentiation by targeting *Ep300* (Hu et al., 2015), suggesting that the function of miR-132 may depend on the type of stimulation or cells.

miR-195-5p is downregulated in hPDLCs under cyclic tensile strain and on the tension side of the OTM mouse model (Chang et al., 2017). miR-195-5p inhibits osteogenesis by directly targeting *Wnt3a* and *Bmpr1a* (Chang et al., 2017). Similarly, miR-503-5p, miR-146a, and miR-34a are decreased in tension force-stimulated MSCs (Liu et al., 2017; Meng et al., 2021). miR-146a and miR-34a inhibit PDLSC osteogenesis, probably by targeting CUGBP Elav-like family member 3 (*CELF3*) (Meng et al., 2021). miR-503-5p inhibits *Runx2* and *Alp* expression, leading to fewer osteoblasts and reduced bone formation on the tension side of the OTM mouse model (Liu et al., 2017). miR-138 is another important mechanosensitive miRNA in bone metabolism (Brito et al., 2023). In stretched BMSCs, the expression of *FAK* and tension-induced osteogenesis are increased, likely due to decreased levels of miR-138, which specifically binds to *PTK2* (the gene encoding *FAK*) (Wu J. et al., 2018a).

PDLCs can regulate osteoclastogenesis under mechanical load by secreting paracrine factors. Kanzaki et al. demonstrated that compression induces miR-3198 expression while tension reduces it, which is negatively correlated with *OPG* expression (Kanzaki et al., 2019). The regulatory role of miR-3198 on *OPG* may be involved in maintaining the balance between RANKL and OPG, which is crucial for osteoclast differentiation and compression-side bone resorption (Kanzaki et al., 2019). Compression loading decreases miR-26a levels in PDLCs, which also modulates osteoclastogenesis by targeting *Jagged1* and regulating *RANKL* and *IL-6* expression (Zhang et al., 2021). Extracellular matrix (ECM) homeostasis is another important aspect of OTM. The miR-29 family, including miR-29a-3p, miR-29b-3p, and miR-29c-3p, is involved in ECM homeostasis in periodontal tissue (Chen et al., 2015). The expression of the miR-29 family is downregulated under cyclic stretch stimulation and upregulated under compression-force stimulation in PDLCs (Chen et al., 2015). *Col1a1*, *Col3a1,* and *Col5a1*, the major periodontal ligament ECM genes, are negatively correlated with miR-29 expression under stretch or compression stimulation. These findings suggest that miR-29 might participate in ECM remodeling during OTM.

#### 4.1.3 Osteoblasts and cementoblasts

As the effector cells of bone formation, osteoblasts are sensitive to mechanical stimulation (Mai et al., 2013). FSS stimulation rapidly downregulates miR-20a, −21, −19b, −34a, −34c, −140, and −200b in osteoblasts (Mai et al., 2013). Interestingly, after a transient decrease, the expression levels of miR-20a, −19b, and −34a are upregulated in osteoblasts, and this trend persists with the increased osteogenesis under FSS. miR-20a may function as a crux in FSS-induced osteogenesis by targeting *Bambi* and *Smad6*, thereby enhancing *Bmp2* signaling (Peng et al., 2022). FSS-induced downregulation of miR-140-5p may also promote osteoblast proliferation through *Vegfa* and the activation of ERK5 signaling. Mechanical tensile strain upregulates miR-191, -3070, -3077-5p, -3090-5p, and -3103-5p and downregulates miR-33, -218, −466i-3p, and 466h-3p in MC3T3-E1 cells (pre-osteoblastic cells) (Guo et al., 2015; Wang et al., 2015). Another study showed that miR-33-5p in MC3T3-E1 cells is sensitive to different mechanical stimuli. In this study, miR-33-5p is downregulated under microgravity and upregulated under FSS to promote cell differentiation by targeting *Hmga2* (Wang H. et al., 2016a). miR-494-3p inhibits the proliferation of MC3T3-E1 cells by targeting *Fgfr2* and *Rock1* (Iwawaki et al., 2015). *Runx2*, as the most important factor in osteoblast differentiation, is directly targeted by miR-103a (Zuo et al., 2015). Cyclic stretch stimulation downregulates miR-103a, increases *Runx2* expression, and enhances osteogenic differentiation in hFOB1.19 cells. Notably, increased miR-103a levels are correlated with bone loss caused by mechanical unloading (Zuo et al., 2015).

Cementoblasts share the same origin as osteoblasts and are located at the interface between the periodontal ligament and tooth roots. They produce cementum as a protective barrier for the tooth root during OTM (Wang L. et al., 2016b). Several studies have shown that mechanosensitive ncRNAs, including lncRNAs (Liu et al., 2019) and miRNAs (Wang L. et al., 2016b), participate in cementoblastogenesis. After 18 h of cyclic tensile stress stimulation, 71 miRNAs are upregulated and 32 miRNAs are downregulated in cementoblasts. The MAPK, Wnt, and TGFβ/BMP-Smad signaling pathways are associated with these changes (Wang L. et al., 2016b). Further analysis revealed that miR-146b-5p, the most upregulated miRNA after 6 h of stimulation, enhances cementoblastogenesis under tensile stimulation by targeting *Smad4* (Wang L. et al., 2016b).

#### 4.1.4 Clastic cells

Monocyte-derived clastic cells, such as osteoclasts, cementoclasts, and odontoclasts, play a crucial role in bone and tooth root resorption during OTM. M-CSF, RANKL, and OPG are crucial factors during osteoclastogenesis (Rody et al., 2001; Li et al., 2021). As mentioned above, miR-21 regulates not only osteoblastogenesis but also osteoclastogenesis (Chen N. et al., 2016a). In a *miR-21−/−* mouse model, *miR-21* deficiency inhibits compression-side alveolar bone resorption and osteoclastogenesis, particularly during OTM. Interestingly, the inhibitory effect of *miR-21* deficiency is not associated with decreased *Rankl* levels (Hu et al., 2017); *Rankl* levels are actually increased in *miR-21−/−* mice. *Pdcd4* might play a key role in the inhibition of osteoclastogenesis (Chen N. et al., 2016a). In the rapid maxillary expansion experiment, the expression ratio of *OPG/RANKL* is decreased in the midpalatal suture of *miR-21−/−* mice, which promotes osteoclastogenesis (Li M. et al., 2020a).

The abnormal formation of cementoclasts and odontoclasts during OTM may result in unfavorable root resorption (Jiang et al., 2021). Jiang et al. found that the level of miR-155-5p is decreased in the gingival crevicular fluid of patients with orthodontic root resorption, while the levels of *CXCR2* and *dentin phosphoprotein* (*DPP*) are increased depending on the severity of root resorption (Jiang et al., 2021). miR-155-5p directly inhibits the expression of *Cxcr2* and suppresses the differentiation of RANKL-induced cementoclasts/odontoclasts. It also reduces the indicator enzymes of mineral resorption (CAII, MMP-9, and cathepsin K) in RANKL-induced cementoclast/odontoclast progenitor cells (RAW264.7 cells), suggesting the protective role of miR-155-5p in orthodontic root resorption (Jiang et al., 2021).

#### 4.1.5 Chondrocytes

The remodeling of the condylar cartilage is closely related to OTM, and improper orthodontic force may lead to cartilage inflammation and degradation (Tanaka et al., 2008). Chondrocytes are located in the condyle of the temporomandibular joint (TMJ) in humans and also in the mid-palatal suture in mice. Mechanical loading from the unilateral anterior crossbite (UAC) leads to severe cartilage degeneration and loss of the extracellular matrix in the temporomandibular joint osteoarthritis (TMJOA) model. Compared with the control group, the expression level of miR-21-5p is upregulated in the TMJOA group (Ma et al., 2020). *miR-21-5p* knockout reduces both TMJOA progression and the levels of inflammatory molecules, including MMP-13 and VEGF. Further studies showed that miR-21-5p is involved in the IL-1β-induced degradation of the extracellular matrix of the condylar cartilage by targeting *Spry1* and promoting angiogenesis through the ERK/MAPK signaling pathway (Ma et al., 2020).

In addition to the above-mentioned cells, neurons, immune cells, and epithelial cells also participate in maxillofacial bone remodeling and OTM (Li et al., 2021). Macrophages are important regulators of osteogenesis, and miR-125a-5p promotes M2 macrophage polarization and facilitates osteogenesis by targeting *ETV6* (He et al., 2021b). The balance between RANKL and OPG in T cells is also associated with miR-21-mediated OTM (Wu et al., 2020). Notably, a cyclic stretch causes the loss of tight junction integrity in rat alveolar epithelial cells and increases epithelial permeability with 42 differentially expressed miRNAs. Among them, the inhibition of miR-466d-5p and miR-466f-3p leads to the recovery of epithelial permeability (Yehya et al., 2012).

Mechanosensing is the basis of maxillofacial bone remodeling and OTM. The aforementioned studies on mechanosensitive miRNAs expand our understanding of cell mechanobiology and provide potential therapeutic approaches to regulate these processes.

### 4.2 miRNAs in the mechanical load-induced hypoxic microenvironment

The mechanical load-induced hypoxic microenvironment also contributes to OTM. A local hypoxic microenvironment promotes the release of cytokines and recruits immune cells, resulting in angiogenesis, osteoblastogenesis, and osteoclastogenesis (Dandajena et al., 2012; Li et al., 2021). Hypoxia-inducible factor-1α (*HIF-1α*) is the most important and sensitive factor induced by hypoxia (Dandajena et al., 2012). Increased *HIF-1α* levels in PDLCs might play a synergistic but independent role under mechanical loading (Li M. L. et al., 2016c). The orthodontic force rapidly induces HIF-1α expression, which peaks on day 7 and then decreases over time (Zhang X. et al., 2019b). This kinetic change is correlated with the expression pattern of miR-21, suggesting a potential association between HIF-1α and miR-21. An *in vitro* study revealed that miR-21 could increase *HIF-1α* levels and several osteogenic markers, such as *OPN*, *RUNX2,* and *ALP*, resulting in osteoblastogenesis and new bone formation (Zhang X. et al., 2019b). However, the effects of hypoxia on osteogenic differentiation are still controversial; in another study, Ye et al. performed ceRNA network analysis in PDLSCs under hypoxia. They selected 5 miRNAs, 21 circRNAs, 262 lncRNAs, and 5 mRNAs for ceRNA network construction. Further analysis showed that the lncRNA FTX/circRNA FAT1/hsa-miR-4781-3p/SMAD5 axis and circRNA LPAR1/hsa-miR-342-3p/ADAR axis were closely related to the inhibited osteogenic differentiation of PDLSCs under hypoxia (Ye et al., 2021). In addition to its impact on osteogenesis, hypoxia can also inhibit PDLSC proliferation and induce apoptosis. This process is mediated by decreased miR-646 and increased IGF-1 levels, and the upregulation of miR-646 rescues PDLSCs from growth inhibition and apoptosis (Yang et al., 2018).

### 4.3 miRNAs in intercellular communication during bone remodeling

Intercellular communication plays a crucial role in bone remodeling. Exosomes and other EVs are the primary vehicles for transporting miRNAs (Liu et al., 2018). After the fusion of EVs with the target cell membrane, miRNAs enter the recipient cells and exert regulatory functions. Here, we introduce miRNAs in osteocyte-, osteoblast-, osteoclast-, and MSC-derived EVs and focus on the multidirectional communication between these cells.

#### 4.3.1 Osteocyte-derived EVs

After being exposed to mechanical strain, osteocyte-derived exosomes show an increased ability to induce the proliferation and osteogenic differentiation of PDLSCs (Lv et al., 2020). This effect is driven by the increased level of miR-181b-5p in stimulated exosomes, which targets the PTEN/AKT signaling pathway and alleviates the inhibition of PDLSC proliferation induced by inflammation (Lv et al., 2020). Myostatin stimulation downregulates miR-218 in osteocyte exosomes, leading to the inhibition of osteoblast differentiation by targeting the Wnt signaling pathway, suggesting the mediating role of osteocytes in muscle–bone communication (Qin et al., 2017). Osteocyte exosomes are also released into the blood to influence miRNA levels in plasma, suggesting that osteocyte exosomes may act as a systemic regulator. However, the specific function of these miRNAs is still unclear (Sato et al., 2017).

#### 4.3.2 Osteoblast-derived EVs

Osteoblasts sense mechanical stimulation and regulate the function of multiple bone-related cells. The miRNAs in osteoblast exosomes play a significant role in osteoblast–osteoblast, –MSC, –osteoclast, and –osteocyte communication. miR-30d-5p and miR-140-3p secreted from mineralizing MC3T3-E1 cells can inhibit osteoblast differentiation by targeting *Runx2* and *Bmp2* (Zhang et al., 2011; Hwang et al., 2014). Several exosomal miRNAs from MC3T3-E1 (miR-6769b-5p, -7668-3p, -7044-5p, and -874-3p) participate in the osteogenesis of MSCs by targeting the *Axin1* and Wnt signaling pathways (Cui et al., 2016). The communication and interaction between bone-building cells (osteoblasts) and bone-absorbing cells (osteoclasts) are crucial during OTM. Osteoblasts can regulate osteoclast differentiation through the exosomal miR-503-3p/Hpse axis. The upregulation of exosomal miR-503-3p inhibits osteoclast formation (Wang Q. et al., 2021a).

#### 4.3.3 Osteoclast-derived EVs

Osteoclasts are sensitive to various mechanical stimulations (Rody et al., 2001; Wang et al., 2022). The compression load attenuates miR-146a-5p expression in osteoclast exosomes, which significantly promotes the proliferation and function of endothelial cells and enhances angiogenesis by targeting *adiponectin*. This mechanism might contribute to orthodontic force-induced angiogenesis and osteoclastogenesis (Wang et al., 2022). Osteoclast-derived EVs can also inhibit osteoblast activity through miR-214 (Sun et al., 2016). miR-214-enriched exosomes from osteoclasts specifically inhibited osteoblast function by targeting *Atf4*, resulting in reduced *Alp*, *Bglap*, and *Col1α1* mRNA levels. The inhibition of exosome release can rescue osteoblast dysfunction and osteoporosis *in vitro* and *in vivo* (Li D. et al., 2016; Sun et al., 2016).

#### 4.3.4 MSC-derived EVs

MSC-derived exosomes have been extensively investigated in bone repair and immune regulation (Lotfy et al., 2023). After osteogenic induction, exosomes from PDLSCs promoted the osteogenic differentiation of BMSCs with altered miRNA profiles. miR-122-5p, -142-5p, -25-3p, and -192-5p are significantly increased, while miR-125b-5p, let-7b-5p, and miR-100-5p are decreased compared to exosomes from undifferentiated PDLSCs (Liu et al., 2020). The compression load can promote type H vessel formation, enhancing bone formation and remodeling (Peng et al., 2020). This process might be regulated by miR-214-3p in BMSC exosomes. The downregulation of exosomal miR-214-3p increases the *VEGF* levels and promotes angiogenesis (Wang et al., 2020), which may also contribute to orthodontic force-induced angiogenesis and bone remodeling.

**FIGURE 4:**
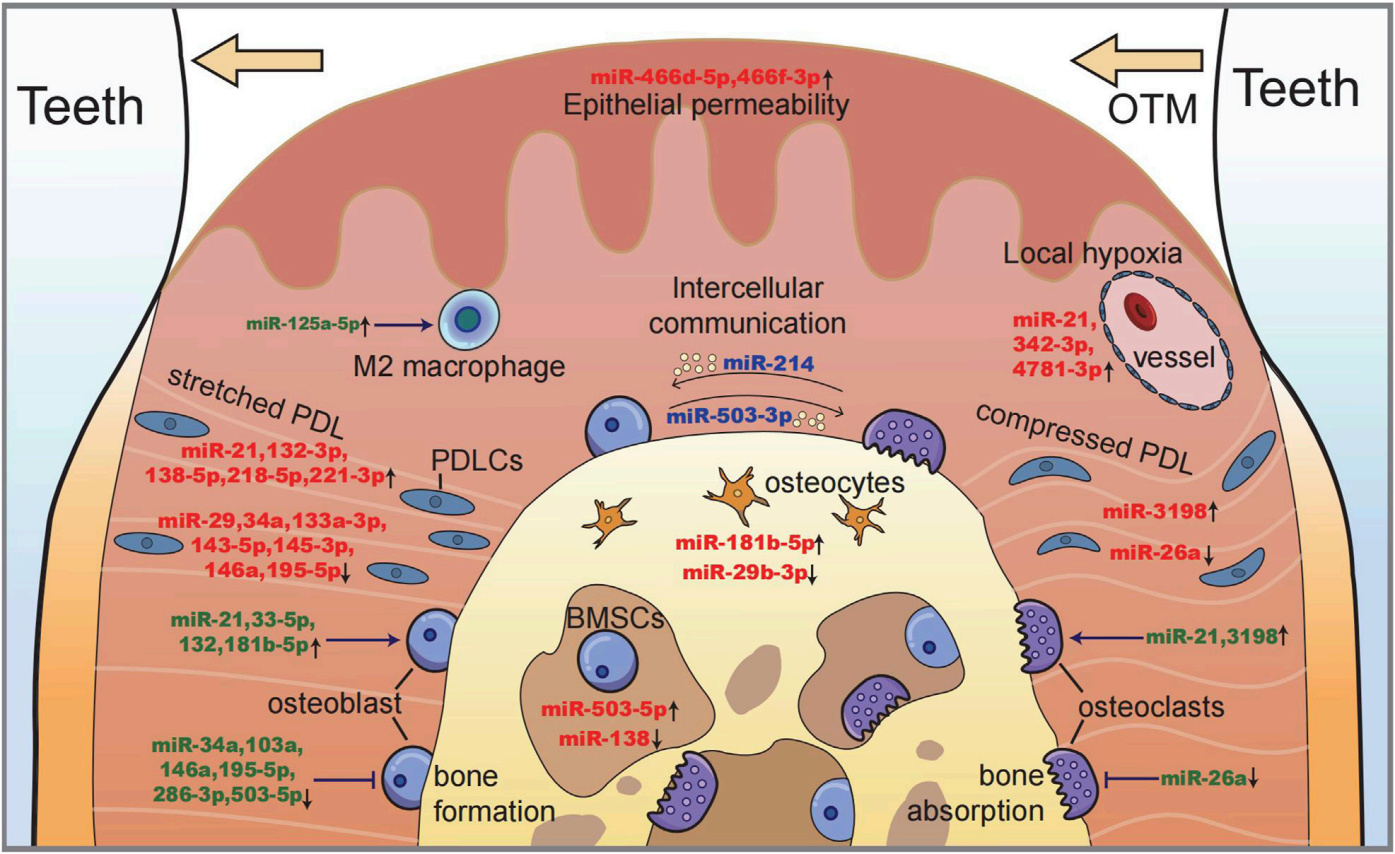
Illustration of miRNAs in maxillofacial bone remodeling during OTM. Along with tooth moving, bone deposits on the tension side (left) with a stretched periodontal ligament and osteoblastogenesis, while bone resorption occurs on the compression side (right) with the local hypoxic area and osteoclastogenesis. miRNAs function as (i) the sensors of mechanical stimulation (red), (ii) the regulators of cell proliferation and differentiation (green), and (iii) the intercellular messengers between different cells (blue).

In summary, miRNAs that participate in mechanosensing and intercellular communication are shown in Figure 4, which illustrates the processes of bone remodeling during orthodontic tooth movement.

## 5 Therapeutic potential of miRNAs for orthodontic treatment

### 5.1 miRNAs as biomarkers

The potential biomarker miRNAs in maxillofacial bone malformation and malocclusion are summarized in Table 1. miRNA levels in the periodontal tissue can be monitored during orthodontic treatment due to its sensitivity to mechanical stimulation (Kapoor et al., 2021). Gingival crevicular fluid (GCF) is the serum exudate in the gingival crevice that contains bioactive components. The level of miR-29 in GCF rapidly increases after canine retraction and continues to increase for 6 weeks after the treatment (Atsawasuwan et al., 2018). The miR-34 level is gradually downregulated in GCF from both the tension and compression sides and returns to baseline after 12 weeks (Zhang B. et al., 2020a). The miR-155-5p level in GCF is correlated with the severity of root resorption. Specifically, the level of miR-155-5p is two times higher in healthy individuals than in the severe root resorption group, indicating its potential use as a biomarker of root resorption during OTM (Jiang et al., 2021). Several miRNAs, including miR-4291, -1245b-3p, and −1825, have been linked to peri-implantitis in patients receiving miniscrew implants during orthodontic treatment (He et al., 2021a). These findings suggest that miRNAs may be used to monitor the risks associated with orthodontic treatment.

### 5.2 miRNA in accelerating bone remodeling

Orthodontic treatment typically lasts 2–3 years. Prolonged treatment may increase the risk of caries, gingival recession, and root resorption (Nimeri et al., 2013). Several surgical and physical approaches, such as periodontal accelerated osteogenesis orthodontics (PAOO), vibration, and pulsed electromagnetic fields (PEMFs) (Nimeri et al., 2013), have been used to accelerate bone remodeling and tooth movement. The miR-21 level is upregulated after surgical stimulation during PAOO treatment and is correlated with accelerated tooth movement. Using the artificial agonist of miR-21 (AgomiR-21) can significantly increase the moving rate, while the antagonist of miR-21 (antagomiR-21) neutralizes the effect of PAOO (Zhang Y. et al., 2020c). Physical stimulation can also induce changes in the expression of specific miRNAs, increasing osteogenic differentiation and promoting bone formation, thereby accelerating tooth movement (De Mattei et al., 2020; Yu et al., 2020). Using N-acetyl-L-leucine-modified polyethylenimine (N-Ac-l-Leu-PEI) as a carrier for miR-34a, the delivery of miR-34a promoted alveolar bone remodeling and OTM in a rat model (Yu et al., 2017). Although this new delivery system showed low toxicity and high biocompatibility compared with traditional carriers, its effect on humans remains unclear. These studies indicate the bridging function of miRNAs between various stimuli and OTM, suggesting that miRNAs can be used to accelerate orthodontic treatment.

### 5.3 miRNAs in periodontal homeostasis

miRNAs play an important role in maintaining periodontal homeostasis (Luan et al., 2017). Subperiosteal injection of agomir-218 (the agonist of miR-218) decreases pro-inflammatory cytokines and MMP9 levels in a periodontitis rat model (Guo et al., 2019). By locally injecting a miR-200c-containing vector that is incorporated with branched polyethylenimine (PEI), the pro-inflammatory and osteoclastogenic markers IL-6, IL-8, and IFRD1 are significantly downregulated with less alveolar bone loss in periodontitis rats (Akkouch et al., 2019b). The osteogenic potential of miR-21 has also been employed to promote alveolar bone regeneration. Research shows that miR-21-loaded Bio-Oss particles enhance the osteogenesis of stem cells and the regeneration of alveolar bones compared with conventional particles (Li X. et al., 2020b). This finding not only demonstrates the protective effects of miRNAs in periodontitis but also shows the feasibility of using miRNA delivery systems for periodontitis treatment. Exosomes are another ideal vehicle for delivering therapeutic miRNAs to treat periodontitis due to their biocompatibility, stability, and low immunogenicity (Zheng et al., 2019). In addition, exosomes can be modified by adding specific miRNAs and targeting peptides to enhance their delivery capacity (Xitong and Xiaorong, 2016). However, miRNA therapy for periodontitis is still in its early stages because we lack reliable ways to safely and effectively deliver these molecules to patients.

## 6 Future prospects and challenges

Since its discovery in 1993, miRNA has redefined the canonical central dogma of gene expression and regulation (Lee et al., 1993). miRNAs participate in bone modeling and remodeling, and their pathogenesis holds great potential for the treatment of diseases.

miRNAs involved in congenital orofacial clefts or other developmental anomalies may serve as tools for the better diagnosis and prevention of diseases (Iwaya et al., 2023). Different cell types have distinct expression profiles of miRNAs. Moreover, the roles of some miRNAs are controversial, with some exhibiting contrasting functions in osteoblast and osteoclast differentiation (Nakasa et al., 2011; Cheung et al., 2014). Although molecular mimics or inhibitors of miRNAs are widely used to study their biological effects, understanding how miRNAs affect development and health remains challenging. One reason for this complexity is the intricate “many-to-many” relationship between miRNAs and their target genes, which could result in conflicting outcomes under different conditions. Second, the function of miRNAs is also regulated by other ncRNAs. These complex regulatory networks increase the difficulty of research. Until now, most studies have been limited to *in vitro* and experimental mouse models, which may not accurately reflect how miRNAs work in humans. Therefore, more research is needed to understand how miRNAs affect human health and disease.

Utilizing the plasticity of bone for orthodontic or orthopedic treatment has been documented for a long time. Understanding the regulatory mechanisms of miRNAs is important for developing drugs or physical methods to promote fracture healing, distraction osteogenesis, and OTM. While miRNAs show potential for treating bone diseases, translating this into actual clinical practice requires extensive research. Future studies should investigate the optimal dosage and timing for clinical applications and develop effective and safe delivery systems. Moreover, the intricate ways in which miRNAs respond to various stimuli and their diverse functions across different cells need to be further investigated.

## 7 Scope statement

Due to the high incidence of maxillofacial bone malformations and malocclusion, studying their pathogenesis and treatments are of great importance. As the major epigenetic regulators, microRNAs (miRNAs) have been proved to regulate facial development. However, a comprehensive illustration of the mechanisms throughwhich miRNAs regulate maxillofacial bone malformations and their therapeuticpotential is still lacking. Based on these questions, we summarize the roles of miRNAs in maxillofacial bone modeling and remodeling, which represent the two main stages in bone biology. In the modeling section, the correlations between miRNAs and maxillofacial bonemalformations and malocclusion are summarized. In the context of remodeling, miRNAs serve as (i) sensors of mechanical stimulation, (ii) mediators of intercellular communication. We also underscore the role of miRNAs in soft tissues (such as theperiodontal ligament) as the key to understand the high mechanosensitivityof maxillofacial bones compared with limb bones. We believe this paper is relevant tothe scope of Frontiers in Cell and Developmental Biology and will be valuable for researchers in this field.
